# Sustainable Development Goals, Nurturing Care and child development monitoring: educational perspectives in nursing

**DOI:** 10.1590/0034-7167-2024-0248

**Published:** 2025-03-14

**Authors:** Débora Falleiros de Mello, Flávia Azevedo Gomes-Sponholz, Luciana Mara Monti Fonseca, Carolina Beltreschi Bardivia

**Affiliations:** I Universidade de São Paulo. Ribeirão Preto, São Paulo, Brazil

**Keywords:** Child Development, Sustainable Development, Child Care, Primary Health Care, Nursing Education, Desarrollo Infantil, Desarrollo Sostenible, Cuidado Infantil, Atención Primaria de Salud, Educación en Enfermería

## Abstract

**Objectives:**

to list educational elements in nursing with a focus on monitoring child development in the context of the Sustainable Development Goals and Nurturing Care.

**Methods:**

qualitative report on an educational perspective for nursing undergraduates. The following stages emerged: key conceptual dimensions, simulated situation, clinical field, case study and assessment.

**Results:**

educational elements were listed regarding child development monitoring prioritizing home visits. Contents and practices were anchored in scientific evidence from Nurturing Care and Sustainable Development Goals in their peculiarities of promoting child development, health and well-being, eradicating poverty and hunger, early childhood education and reducing inequalities.

**Final Considerations:**

the educational perspective presents itself as a triangulation between theoretical knowledge, simulation and clinical environment, with challenges in the face of circumstances, strengths and weaknesses in simulated situations and with families with children, allowing us to recognize the potential of existing practices and the barriers to be broken.

## RESUMEN

**Objetivos:**

enumerar elementos educativos en enfermería con enfoque en el seguimiento del desarrollo infantil en el contexto de los Objetivos de Desarrollo Sostenible y el *Nurturing Care*.

**Métodos:**

informe cualitativo desde una perspectiva educativa para estudiantes de enfermería. Se presentan los pasos: dimensiones conceptuales clave, situación simulada, campo clínico, estudio de caso y evaluación.

**Resultados:**

se enumeraron elementos educativos en relación al seguimiento del desarrollo infantil, priorizando las visitas domiciliarias. Los contenidos y prácticas estuvieron anclados en la evidencia científica de los *Nurturing Care* y los Objetivos de Desarrollo Sostenible en sus peculiaridades de promover el desarrollo, la salud y el bienestar de los niños, erradicar la pobreza y el hambre, la educación de la primera infancia y reducir las desigualdades.

**Consideraciones Finales:**

la perspectiva educativa se presenta como una triangulación entre conocimientos teóricos, simulación y entorno clínico, con desafíos dadas las circunstancias, fortalezas y debilidades en situaciones simuladas y con familias con niños, proporcionando reconocimiento del potencial de las prácticas existentes y de las barreras que deben romperse.

## INTRODUCTION

The first years of life, especially the period between birth and the age of 6, is called early childhood. It is an important period of human development, as it is the basis for the formation and improvement of complex skills related to attention, memory, problem-solving ability and critical judgment^(1)^.

The United Nations 2030 Agenda, composed of 17 Sustainable Development Goals (SDGs), includes guidelines for promoting health and child development related to increasing health and well-being, eradicating poverty and hunger, and reducing inequalities^(2)^. Furthermore, ensuring universal access to healthcare services is considered essential for child development and for child protection and care^(2)^. Within the SDGs, the importance of investing in early childhood development for social cohesion, economic growth and construction of peaceful and inclusive societies was recognized, and efforts have been directed to reduce mortality and, above all, promote the full development of all children^(3)^.

Indicators that aim to achieve adequate development in early childhood focus on the dimensions of good health, adequate nutrition, safety and security, responsive caregiving, and opportunities for early learning, which make up the Nurturing Care domains as a network of care to promote child comprehensive development^(1)^. In light of the SDGs, in many countries and in Brazil, the countdown to 2030 points to weak indicators, such as the lack of data on children with adequate development, appropriate stimulation at home, positive discipline, responsive care, among others^(3)^.

Scientific evidence expresses the relevance of child development surveillance in monitoring conditions that affect human development for their early identification and implementation of appropriate interventions^(1)^. Nurses play an essential role in providing shared opportunities with families to develop child care skills, expand understanding of child development, and promote health and conscious change^(4)^.

In the face of contemporary challenges, it is worth highlighting that early childhood development is still a largely neglected topic in low and middle-income countries, where children face many adversities in reaching their potential^(1,3)^. In the context of nursing care in Primary Healthcare (PHC), interventions in line with the SDG targets with families and communities become fundamental in clinical practice, particularly in home visits (HVs) and consultations in childcare and puerperium, contributing greatly to good health conditions and indicators^(5)^. In the field of maternal and child nursing training, it is vital to provide undergraduate students with flexible learning experiences, varied strategies and opportunities, simulation and expansion of clinical practice^(6)^, enabling the construction of skills to meet children’s, women’s and families’ needs from the perspective of the SDGs^(7)^.

## OBJECTIVES

To list educational elements in nursing with a focus on monitoring child development in the context of the SDGs and Nurturing Care.

## METHODS

### Ethical aspects

As this is a descriptive study and does not involve human beings, the research was not submitted to a Research Ethics Committee, in accordance with Resolution 466/2012.

### Report stages

This descriptive and qualitative report followed the guidelines adapted from the Standards for Reporting Qualitative Research (SRQR) checklist to increase transparency and rigor in qualitative research synthesis.

The educational perspective presented here focuses on monitoring child development, prioritizing HV, and is based on the stages (i) key conceptual dimensions, (ii) simulated situation, (iii) clinical field and (iv) case study and written assessment, anchored in scientific evidence of the Nurturing Care approach^(1)^ and the SDG^(2,3)^. The aforementioned stages are part of a 150-hour course in an undergraduate nursing course (bachelor’s degree lasting eight semesters), taught in the fourth semester for 80 students.

The key conceptual dimensions stage is carried out through oral presentation in the classroom and through the provision of reading and study materials via e-disciplines. This is an educational technology system based on the Moodle (Modular Object-Oriented Dynamic Learning Environment) virtual learning environment customization, called e-disciplines, created to support the disciplines of this university.

The simulated situation stage is centered on HV, offered in subgroups of up to 20 students, in a teaching laboratory with a scenario characterized as a simulated house, a medium-fidelity baby mannequin and objects. The activity, unique for each subgroup, consists of a hybrid, high-fidelity simulation scenario, lasting three hours, with 30 minutes of prebriefing, 50 minutes of simulated scene and one hour and 40 minutes of debriefing. In prebriefing, facilitating professors and students sign a contract for a safe and controlled learning environment, and receive instructions on the activity, the scenario, the learning objectives, the mannequin, the role-playing director, and the summary of a simulated case. They then discuss the case and organize the activity presentation among peers, without the presence of professors. Afterwards, they carry out the simulated activity itself, with the participation of two students and a role-playing director (a graduate nurse in the role of a baby’s caregiver), whereas the other students and professors observe the scene without making any comments.

After the simulated HV, the debriefing used was structured and supported - G.A.S debriefing^(8)^. Initially, the first phase is to gather (Gather) emotional and descriptive aspects (how did you feel while carrying out the activity? How do you describe the scenario you experienced?); the second phase is to analyze (Analyze) the facts and establish bridges with the theory (what did you do correctly? What would you do differently? What points could be improved?); and the last phase is to summarize (Summarize), bringing synthesis about the facts and final reflection (what will you take to your learning?).

The moments of group theorizing and reflection are triggered and highlight aspects of: communication with caregiver (role played by the director); baby observation; interaction with baby; specific elements of child development and family relationships; data on child health, nutrition, child sleep, vaccination, support network, decision-making, among others; guidance on care routines and protection and safety measures in everyday home life; and focus on comprehensive care.

The clinical field stage is carried out in a health unit with a Family Health Strategy, with a workload of eight hours specifically dedicated to HV activities based on child development monitoring. In the clinical field, subgroups of five students carry out activities in an in-person HV, with each two or three students accompanied by a community health worker and a professor. The activities consist of: recognizing unit and health team; reading and analyzing medical records; organizing and carrying out HV based on a semi-structured script; discussing and reflecting on the clinical practice performed; making nursing notes in medical records; and summarizing for the subsequent preparation of the case study.

The last stage consists of a case study, developed with empirical material learned in the clinical field, carried out in pairs or trios of students, entered into a file in e-disciplines. Subsequently, it consists of a cognitive assessment with multiple-choice questions, with ten questions and four alternatives in each, applied in person and individually.

The individual assessment of each student and group of students is, throughout the discipline, diagnostic, formative and summative, carried out with the other activities. It is worth mentioning that, during the aforementioned discipline, there are other clinical fields and workloads in addition to those detailed here. Furthermore, in this educational institution, students are introduced to the topic of HV in health from the first semester of the first year of the undergraduate nursing course.

### Justification for the report

The educational perspective arose from experiences of professors at a Brazilian nursing school who identified the need for greater connection between theoretical and practical elements and the inclusion of simulation to enhance the teaching-learning process for undergraduate nursing students, given the relevance of monitoring child development through the Nurturing Care^(1)^ domains, the SDGs^(2)^ and the role of nursing in this field^(4-6)^.

## RESULTS

Chart 1 provides a consolidation of educational stages, focusing on monitoring child development and prioritizing HV, in a theoretical-practical sequence.

**Chart 1:** Summary of educational stages related to undergraduate nursing education with a focus on monitoring child development in the context of Nurturing Care and the Sustainable Development Goals

Key conceptual dimensions	Simulated situation	Clinical field	Case study and assessment
- Health surveillance in PHC, Nurturing Care and SDGs. - Brazilian National Policy for Comprehensive Child Healthcare (PNAISC, *Política Nacional de Atenção Integral à Saúde da Criança*). - Essential needs for the growth and development of children. - Child health record. - Breastfeeding and healthy complementary feeding. - Promotion of child development and the role of parenting. - Prevention of accidents in childhood. - Contexts of violence against women and children. - Children’s rights.	HV to family with child. - Clinical case: mother, 28 years old, one baby, vaginal delivery, without complications. He was born blushing, cried and was placed skin-to-skin immediately after birth. They were discharged the next day and are staying at home. The baby is 9 months old and has never been sick. He is eating well and is breastfeeding. A community worker who visits the family brought to the team that the mother has concerns about the baby’s growth and adequate development, that she has not been monitoring the baby at the health unit and is unsure whether to put him in daycare. - Simulation objectives: conduct a simulated HV; identify and provide guidance on child development, health monitoring, feeding, vaccination, prevention of childhood accidents; establish communication and appropriate guidance. - Discussion and reflection. - e-disciplines: nursing notes on simulated HV.	Activities in healthcare services in PHC. Objectives: recognize the purposes of the health unit and the role of the *Sistema Único de Saúde* (SUS, Brazilian Health System); identify aspects of health policy for children and women; to conduct in-person HV; describe the composition and sociodemographic conditions of the family; identify and provide guidance on child development, health monitoring, nutrition, vaccination, and prevention of childhood accidents; establish appropriate communication and guidance. - Discussion and reflection. - Medical records: nursing notes on in-person HV.	Case study based on a script Objectives: describe sociodemographic data, birth history and health monitoring; summarize home environment conditions, its surroundings and support network; characterize how the child is and what happens to the child in their daily life (general condition, breastfeeding, food, sleep, rest, hygiene, eliminations, complications and illnesses); detail parental interaction methods, games, reading children’s books, use of television/cell phone, support network, daycare, among other aspects; highlight how parental caregivers consider how the child is developing; list positive and vulnerable aspects of child health; point out priority points of a child healthcare and monitoring plan. - Assessment in test format (statements containing concept, applicability of knowledge, not isolated facts of answers).

*PHC - Primary Healthcare; SDG - Sustainable Development Goals; HV - home visits.*

Educational elements were selected to guide child development monitoring in the HV action in child health nursing, with the articulation between key concepts, simulation and clinical field, seeking to provide content and practices based on scientific evidence related to Nurturing Care and the SDGs.

The Nurturing Care approach is important for HV practice with a focus on promoting child development, centered on good health, adequate nutrition, safety and security, responsive caregiving and opportunities for early learning, which forms the basis for early childhood. Thus, students have opportunities to know, identify, intervene and reflect on early childhood development surveillance, articulating good health (monitoring growth and development, analysis of the growth chart and child development milestones, use of child’s card), adequate nutrition (support, encouragement and assessment of breastfeeding, analysis of complementary feeding), safety and security (analysis of proximal and community environment), responsive caregiving (analysis of performance and interaction of parental caregivers and the support network) and opportunities for early learning (analysis of appropriate stimuli for child development, access to early childhood education) priority aspects.

Within the scope of the SDGs, the focus is on goals 1, 3, 4 and 10. Students have the opportunity to recognize and reflect on the care circumstances that are favorable or vulnerable to the health and development of children and their families.

Regarding goal 1 (no poverty), when experiencing nursing care in HV, they come into contact with social conditions, available resources and their adequate or weakened use, seeking to understand socioeconomic situations and social determinants, and intervene to reduce health disparities. Goal 3 (good health and well-being) is more directly understood by students, visualizing the vital needs for children and families and carrying out nursing care with actions to promote health, prevent injuries, safety measures, well-being and qualified access to health. For goal 4 (quality of education), contact with parental caregivers in HV allows exploring the types and choices of non-parental care for young children, with an emphasis on access to daycare and preschool as well as providing knowledge of the possibilities of intersectoral actions between healthcare service teams and early childhood education institutions. In relation to goal 10 (reducing inequalities), the approach to social and health inequalities is triggered in the experience of nursing care in HV related to seeking access to quality of life and basic needs for promoting full development, healthcare and preventing diseases.

## DISCUSSION

The educational perspective presented brings a chain of stages, and its theoretical-practical articulation allows for back and forth to explore and deepen knowledge as well as providing students with simulated and in-person experiences, discussions and timely reflections. The stages of this educational perspective seek to bring to light enriching elements for training future nurses.

Healthy early childhood benefits from the Nurturing Care approach and attention to the SDGs, given the scarcity of data related to childhood development problems. In child development surveillance, child healthcare, parenting practices, early childhood education and health promotion in infant and young child care are of great relevance to achieving adequate development in early childhood, requiring investment and resources^(1)^.

Nursing has a shared responsibility to make contact with children and their families a window of opportunity, with interventions in light of the SDGs^(7)^, considering the countdown to 2030, to contribute to a facilitating environment, with protection and safety, and guarantee of effective laws and public policies^(1,3)^. Monitoring child development therefore represents a promising action to detect problems, understand difficulties and seek to meet health needs for promotion and protection in early childhood.

Parental caregivers have many tasks to perform care and understand information, which can be complex, new, specific and/or uncertain, making parental knowledge about child development and its benefits for the comprehensive child care essential for childhood^(9)^. Dialogue with nurses, such as nursing care in home visits, is considered by parental caregivers to promote openness and sensitivity, identify needs and clarify doubts, from a perspective of involvement and participation^(4)^. A study shows that, through a contextually relevant, equitable and accessible practice of attention to children’s and adolescents’ needs, nurses contribute greatly to the progress of achieving the SDGs, even in the face of many challenges^(7)^.

In terms of nursing training, opportunities during the undergraduate course are essential to build a strong foundation and potential for offering qualified practices. Maternal and child learning experiences in the field of PHC are important for undergraduate students, particularly in simulated and clinical environments. Simulation has peculiarities to complement learning, being pointed out as preparation that increases confidence in practice, and nursing students’ experiences in maternal and child healthcare simulation with structured debriefing have proven effective in learning^(6)^. PHC clinical environments are considered valuable learning opportunities for nursing students, contributing to acquiring skills and developing resilience. It is important to ensure the preparation of the field in which students will be inserted, team reception and close supervision^(10)^.

On the other hand, it is important to highlight the difficulties in universal access to healthcare services in view of the difficulties in providing advanced child healthcare related to the shortage of qualified healthcare professionals^(7)^. Therefore, there is a need for greater integration of SDG targets in both the training and continuing education of nurses, seeking to enable qualified clinical practice in various contexts and environments, with greater attention to social justice, environmental health and the construction of partnerships^(5)^. In this way, possibilities are opened to, at the same time, contribute to the 2030 Agenda and expand the qualification of nurses’ professional profile.

### Study limitations

In the explanation of this educational perspective, all the theoretical and practical dimensions that students experience during the undergraduate course presented here were not detailed. Nor was there a systematized student assessment in relation to the educational proposal, and feedback from all those involved is important to provide feedback on pedagogical paths, and it is necessary to advance and increase studies on teaching-learning skills and processes.

### Contributions to nursing and health

The educational perspective presented points to the relevance of early childhood as an essential phase for child healthcare, when it is more effective to act and less expensive than later seeking to reverse or mitigate the effects of adversity.

In this report, increased educational strategies and improved knowledge were important to bring qualitative elements in favor of learning and discussion about early childhood and child development monitoring. This movement contributes to nursing care, particularly in primary child healthcare settings, in accordance with the precepts of the SDGs, with emphasis on goals 1, 3, 4 and 10.

## FINAL CONSIDERATIONS

This educational perspective highlighted child development monitoring in training nurses as a triangulation between theoretical knowledge, simulation and clinical environment.

In the presented path, emphasis is on providing experiences in simulated and practical situations with families with children in HV, detailing experiences during peer nursing training and learning to recognize strengths, weaknesses and potentialities of existing practices and the barriers to be overcome. Such aspects are important for the vision of different contexts and circumstances in the face of contemporary challenges to achieve the SDGs.
