# Supplementary material for: Sustainable Development Goals, Nurturing Care and child development monitoring: educational perspectives in nursing
Source: Rev Bras Enferm. 2025 Mar 14;78(Suppl 1):e20240248. doi: 10.1590/0034-7167-2024-0248 (PMC11913132; doi:10.1590/0034-7167-2024-0248)
Supplement: Translation

# Translation: Objetivos de Desenvolvimento Sustentável, *Nurturing Care* e vigilância do desenvolvimento infantil: perspectivas educacionais em enfermagem

**DOI:** 10.1590/0034-7167-2024-0248pt

**Authors:** Débora Falleiros de Mello, Flávia Azevedo Gomes-Sponholz, Luciana Mara Monti Fonseca, Carolina Beltreschi Bardivia

**Affiliations:** I Universidade de São Paulo. Ribeirão Preto, São Paulo, Brasil

**Keywords:** Desenvolvimento Infantil, Desenvolvimento Sustentável, Cuidado Infantil, Atenção Primária de Saúde, Educação em Enfermagem

## Abstract

**Objetivos:**

elencar elementos educacionais em enfermagem com foco na vigilância do desenvolvimento infantil no contexto dos Objetivos de Desenvolvimento Sustentável e *Nurturing Care*.

**Métodos:**

relato qualitativo sobre uma perspectiva educacional para graduandos em enfermagem. São apresentadas as etapas: dimensões conceituais-chave, situação simulada, campo clínico, estudo de caso e avaliação.

**Resultados:**

os elementos educacionais foram elencados quanto à vigilância do desenvolvimento infantil priorizando a visita domiciliar. Conteúdos e práticas foram ancorados em evidências científicas do *Nurturing Care* e Objetivos de Desenvolvimento Sustentável em suas peculiaridades de promoção do desenvolvimento das crianças, saúde e bem-estar, erradicação da pobreza e fome, educação infantil e redução das desigualdades.

**Considerações Finais:**

a perspectiva educacional apresenta-se como uma triangulação entre saberes teóricos, simulação e ambiente clínico, com desafios diante das circunstâncias, fortalezas e fragilidades nas situações simuladas e junto às famílias com crianças, proporcionando reconhecer as potencialidades das práticas existentes e as barreiras a serem rompidas.

## INTRODUÇÃO

Os primeiros anos de vida, especialmente o período entre o nascimento e os 6 anos incompletos, é denominado primeira infância. É um período relevante do desenvolvimento humano, por ser a base para a formação e o aperfeiçoamento de habilidades complexas relacionadas à atenção, memória, capacidade de resolução de problemas e juízo crítico^(1)^.

A Agenda 2030 da Organização das Nações Unidas, composta pelos 17 Objetivos de Desenvolvimento Sustentável (ODS), inclui direcionamentos à promoção da saúde e ao desenvolvimento das crianças relacionados ao incremento da saúde e bem-estar, erradicação da pobreza e fome, e redução das desigualdades^(2)^. Ainda, a garantia do acesso universal aos serviços de saúde é colocada como primordial para o desenvolvimento infantil e para a proteção e cuidados com a infância^(2)^. No âmbito dos ODS, foi reconhecida a importância de investir no desenvolvimento na primeira infância para a coesão social, o crescimento econômico e a construção de sociedades pacíficas e inclusivas, e esforços vêm sendo direcionados para reduzir a mortalidade e, sobretudo, promover o desenvolvimento pleno de todas as crianças^(3)^.

Os indicadores que visam atingir um adequado desenvolvimento na primeira infância focam nas dimensões da saúde, nutrição, segurança e proteção, cuidados responsivos, e aprendizagem oportuna, as quais compõem os domínios do *Nurturing Care* como uma rede de cuidados para promover o desenvolvimento integral das crianças^(1)^. Em atenção aos ODS, em muitos países e no Brasil, a contagem regressiva para o ano de 2030 aponta para indicadores frágeis, a exemplo da ausência de dados sobre crianças com desenvolvimento adequado, estimulação apropriada em casa, disciplina positiva, cuidados responsivos, entre outros^(3)^.

Evidências científicas expressam a relevância da vigilância do desenvolvimento infantil no monitoramento das condições que afetam o desenvolvimento humano para a sua identificação precoce e realização de intervenções apropriadas^(1)^. Enfermeiras(os) possuem papel essencial para proporcionar oportunidades compartilhadas com as famílias para o desenvolvimento de habilidades referentes ao cuidado da criança, ampliar as compreensões sobre o desenvolvimento infantil, promover a saúde e mudanças conscientes^(4)^.

No caminho de desafios contemporâneos, cabe destacar que o desenvolvimento na primeira infância ainda é um tema bastante negligenciado nos países de baixa e média renda, onde crianças enfrentam muitas adversidades para o alcance de seus potenciais^(1,3)^. No âmbito do cuidado de enfermagem na Atenção Primária à Saúde (APS), intervenções consonantes às metas dos ODS junto às famílias e comunidades tornam-se fundamentais na prática clínica, particularmente em visitas domiciliares (VDs) e consultas em puericultura e puerpério, contribuindo, sobremaneira, para boas condições e indicadores de saúde^(5)^. No campo da formação em enfermagem materno-infantil, é vital proporcionar aos estudantes de graduação experiências de aprendizagem flexíveis, estratégias e oportunidades variadas, simulação e ampliação da prática clínica^(6)^, possibilitando a construção de competências para atender às necessidades das crianças, mulheres e famílias na perspectiva dos ODS^(7)^.

## OBJETIVOS

Elencar elementos educacionais em enfermagem com foco na vigilância do desenvolvimento infantil no contexto dos ODS e *Nurturing Care*.

## MÉTODOS

### Aspectos éticos

Por se tratar de estudo descritivo e não envolver seres humanos, a pesquisa não foi submetida a Comitê de Ética em Pesquisa, de acordo com a Resolução nº 466/2012.

### Etapas do relato

O presente relato descritivo e qualitativo seguiu as diretrizes adaptadas do *checklist Standards for Reporting Qualitative Research* (SRQR) para ampliar a transparência e rigor na síntese da investigação qualitativa.

A perspectiva educacional aqui apresentada tem foco na vigilância do desenvolvimento infantil, priorizando a VD, e está pautada nas etapas (i) dimensões conceituais-chave, (ii) situação simulada, (iii) campo clínico e (iv) estudo de caso e avaliação escrita, ancorada em evidências científicas da abordagem do *Nurturing Care* ^(1)^ e nos ODS^(2,3)^. As referidas etapas fazem parte de uma disciplina de 150 horas de um curso de graduação em enfermagem (bacharelado com duração de oito semestres), ministrada no quarto semestre para 80 estudantes.

A etapa das dimensões conceituais-chave é realizada por meio de exposição oral dialogada em sala de aula e pela disponibilização de materiais de leitura e estudo via e-disciplinas. Trata-se de um sistema de tecnologia educacional baseado na customização do ambiente virtual de aprendizagem *Moodle* (*Modular Object-Oriented Dynamic Learning Environment*), denominado e-disciplinas, criado para dar apoio às disciplinas desta universidade.

A etapa da situação simulada é centrada em VD, oferecida em subgrupos de no máximo 20 alunas(os), em um laboratório didático com cenário caracterizado como uma casa simulada, um manequim bebê de média fidelidade e objetos. A atividade, única para cada subgrupo, consiste em cenário de simulação híbrido, de alta fidelidade, com a duração de três horas, sendo 30 minutos de *prebriefing*, 50 minutos de cena simulada e uma hora e 40 minutos de *debriefing*. No *prebriefing*, docentes facilitadoras e alunas(os) realizam o contrato para um ambiente seguro e controlado de aprendizagem, e recebem instruções sobre a atividade, o cenário, os objetivos de aprendizagem, o manequim, o encenador e a síntese de um caso simulado. Na sequência, discutem o caso e organizam a apresentação da atividade entre pares, sem a presença de docentes. Após, realizam a atividade simulada em si, com a participação de duas(dois) alunas(os) e um encenador (enfermeira pós-graduanda no papel de cuidadora do bebê), enquanto as(os) outras(os) alunas(os) e docentes observam a cena sem emitir comentários.

Após a VD simulada, o *debriefing* utilizado foi o estruturado e apoiado - *structured and supported* - G.A.S *debriefing* ^(8)^. Inicialmente, a primeira fase é para reunir (*Gather*) aspectos emocionais e descritivos (como se sentiu realizando a atividade? Como descreve o cenário vivenciado?); a segunda fase é para analisar (*Analyze*) os fatos e estabelecer pontes com a teoria (o que fez adequadamente? O que faria diferente? Quais pontos poderiam ser melhorados?); e a última fase é para sumarizar (*Summarize*), trazendo síntese sobre os fatos e reflexão final (o que levará para o seu aprendizado?).

Os momentos de teorização e reflexão em grupo são desencadeados e destacam aspectos sobre: comunicação com a cuidadora parental (papel desempenhado pelo encenador); observação do bebê; interação com o bebê; elementos específicos do desenvolvimento infantil e relacionamento familiar; dados da saúde infantil, alimentação, sono infantil, vacinação, rede de apoio, tomada de decisão, entre outros; orientações quanto às rotinas de cuidados e medidas de proteção e segurança no cotidiano domiciliar; e enfoque da integralidade do cuidado.

A etapa do campo clínico é realizada em unidade de saúde com Estratégia Saúde da Família, com carga horária de oito horas especificamente destinada às atividades de VD baseada na vigilância do desenvolvimento infantil. No campo clínico, subgrupos de cinco alunas(os) realizam as atividades em VD presencial, sendo cada dois ou três acompanhados por agente comunitário de saúde e por uma docente. As atividades são compostas por: reconhecimento da unidade e da equipe de saúde; leitura e análise de prontuário; organização e realização da VD com base em roteiro semiestruturado; discussão e reflexão da prática clínica realizada; anotação de enfermagem em prontuário; e síntese para a elaboração posterior do estudo de caso.

A última etapa é composta pelo estudo de caso, desenvolvido com material empírico apreendido no campo clínico, realizado em duplas ou trios de alunas(os), inserido em arquivo no e-disciplinas. A seguir, é composta pela avaliação cognitiva com questões de múltipla escolha, com dez questões e quatro alternativas em cada uma, aplicada presencial e individualmente.

A avaliação individual de cada aluna(o) e do conjunto deles é, ao longo da disciplina, diagnóstica, formativa e somativa, realizada com as demais atividades. Cabe mencionar que, durante a referida disciplina, há outros campos clínicos e carga horária além das aqui detalhadas. Ainda, nesta instituição de ensino, as(os) alunas(os) têm aproximação à temática da VD em saúde desde o primeiro semestre do primeiro ano do curso de graduação em enfermagem.

### Justificativa para o relato

A perspectiva educacional surgiu das experiências de docentes universitárias em uma escola de enfermagem brasileira que identificaram a necessidade de maior encadeamento entre os elementos teóricos e práticos e da inclusão da simulação para incrementar o processo de ensino-aprendizagem aos estudantes de graduação em enfermagem, tendo em vista a relevância da vigilância do desenvolvimento infantil pelos domínios do *Nurturing Care* ^(1)^, dos ODS^(2)^ e da atuação da enfermagem nesse campo^(4-6)^.

## RESULTADOS

O Quadro 1 traz um consolidado das etapas educacionais, com foco na vigilância do desenvolvimento infantil e priorizando a VD, em um encadeamento teórico-prático.

**Quadro 1:** Síntese das etapas educacionais referentes ao ensino de graduação em enfermagem com foco na vigilância do desenvolvimento infantil no contexto do *Nurturing Care* e dos Objetivos de Desenvolvimento Sustentável

Dimensões conceituais-chave	Situação simulada	Campo clínico	Estudo de caso e avaliação
- Vigilância em saúde na APS, *Nurturing Care* e ODS. - Política Nacional de Atenção Integral à Saúde da Criança (PNAISC). - Necessidades essenciais no crescimento e desenvolvimento da criança. - Caderneta da criança. - Aleitamento materno e alimentação complementar saudável. - Promoção do desenvolvimento infantil e o papel da parentalidade. - Prevenção de acidentes na infância. - Contextos de violência contra mulheres e crianças. - Direitos das crianças.	VD à família com criança. - Caso clínico: mãe, 28 anos, um bebê, parto vaginal, sem intercorrências. Nasceu corado, chorou e foi colocado pele-a-pele imediatamente após o parto. Tiveram alta no dia seguinte e seguem no domicílio. Bebê está com 9 meses e nunca ficou doente. Está comendo bem e está em aleitamento materno. Agente comunitário que visita a família trouxe para a equipe que a mãe refere dúvidas quanto ao crescimento e desenvolvimento adequado do bebê, que não tem realizado acompanhamento do bebê na unidade de saúde e está em dúvida se vai colocá-lo na creche. - Objetivos da simulação: realizar VD simulada; identificar e orientar sobre desenvolvimento infantil, acompanhamento da saúde, alimentação, vacinação, prevenção de acidentes na infância; estabelecer comunicação e orientações adequadas. - Discussão e reflexão. - e-disciplinas: anotação de enfermagem sobre a VD simulada.	Atividades em serviços de saúde na APS. Objetivos: reconhecer as finalidades da unidade de saúde e papel do Sistema Único de Saúde (SUS); identificar aspectos da política de saúde da criança e da mulher; realizar VD presencial; descrever a composição e as condições sociodemográficas da família; identificar e orientar sobre desenvolvimento infantil, acompanhamento da saúde, alimentação, vacinação, prevenção de acidentes na infância; estabelecer comunicação e orientações adequadas. - Discussão e reflexão. - Prontuário: anotação de enfermagem sobre a VD presencial.	Estudo de caso com base em roteiro Objetivos: descrever dados sociodemográficos, histórico de nascimento e seguimento da saúde; sintetizar condições do ambiente domiciliar, seu entorno e rede de apoio; caracterizar como está a criança e o que acontece com a criança no seu dia a dia (estado geral, aleitamento, alimentação, sono, repouso, higiene, eliminações, intercorrências e enfermidades); detalhar modos de interação parental, brincadeiras, leitura de livros infantis, uso de televisão/celular, rede de apoio, creche, entre outros aspectos; destacar como cuidadores parentais consideram como está o desenvolvimento da criança; listar aspectos positivos e vulneráveis à saúde infantil; apontar pontos prioritários de um plano de cuidados e seguimento da saúde infantil. - Avaliação em formato de testes (enunciados contento conceito, aplicabilidade do conhecimento, não fatos isolados de respostas).

*APS - Atenção Primária à Saúde; ODS - Objetivos de Desenvolvimento Sustentável; VD - visitas domiciliares.*

Os elementos educacionais foram selecionados para um direcionamento da vigilância do desenvolvimento infantil na ação de VD em enfermagem em saúde da criança, com a articulação entre os conceitos-chave, a simulação e o campo clínico, buscando proporcionar conteúdos e práticas baseados em evidências científicas referentes ao *Nurturing Care* e aos ODS.

A abordagem do *Nurturing Care* é importante para a prática da VD com foco na promoção do desenvolvimento infantil, centrada na saúde, nutrição, segurança e proteção, cuidados responsivos e aprendizagem oportuna, que constitui a base para a primeira infância. Assim, as(os) estudantes têm oportunidades de conhecer, identificar, intervir e refletir acerca da vigilância do desenvolvimento na primeira infância, articulando os aspectos prioritários de saúde (seguimento do crescimento e desenvolvimento, análise do gráfico de crescimento e dos marcos do desenvolvimento infantil, uso da caderneta da criança), nutrição (apoio, incentivo e avaliação do aleitamento materno, análise da alimentação complementar), segurança e proteção (análise do ambiente proximal e comunitário), cuidados responsivos (análise da atuação e interação dos cuidadores parentais e da rede de apoio) e aprendizagem oportuna (análise dos estímulos adequados ao desenvolvimento infantil, acesso à educação infantil).

No âmbito dos ODS, o enfoque volta-se aos objetivos 1, 3, 4 e 10. As(os) estudantes têm possibilidade de reconhecer e refletir sobre as circunstâncias de cuidado que são favoráveis ou vulneráveis à saúde e ao desenvolvimento das crianças e suas famílias.

Quanto ao objetivo 1 (acabar com a pobreza), ao vivenciarem o cuidado de enfermagem em VD, eles entram em contato com as condições sociais, recursos disponíveis e sua utilização adequada ou fragilizada, buscando compreender as situações socioeconômicas e os determinantes sociais, e intervir para a diminuição de disparidades de saúde. O objetivo 3 (boa saúde e bem-estar) é mais diretamente apreendido pelas(os) alunas(os), visualizando as necessidades vitais para as crianças e famílias e realizando o cuidado de enfermagem com as ações de promoção da saúde, prevenção de agravos, medidas de segurança, bem-estar e acesso qualificado à saúde. Para o objetivo 4 (qualidade da educação), o contato com os cuidadores parentais em VD permite explorar os tipos e escolhas de cuidados não parentais para a criança pequena, com ênfase no acesso à creche e pré-escola, bem como propicia conhecer as possibilidades de ações intersetoriais entre as equipes dos serviços de saúde e instituições de educação infantil. Em relação ao objetivo 10 (reduzir as desigualdades), a abordagem das desigualdades sociais e de saúde é desencadeada na vivência do cuidado de enfermagem em VD relacionada a buscar o acesso à qualidade de vida e às necessidades básicas para a promoção do desenvolvimento pleno, cuidados com a saúde e prevenção de agravos.

## DISCUSSÃO

A perspectiva educacional apresentada traz um encadeamento das etapas, e sua articulação teórico-prática permite idas e vindas para explorar e aprofundar os conhecimentos, bem como possibilitar às(aos) alunas(os) experiências simuladas e presenciais, discussões e reflexões oportunas. As etapas desta perspectiva educacional procuram trazer à tona elementos enriquecedores à formação de futuras(os) enfermeiras(os).

A primeira infância saudável tem ganhos com o enfoque do *Nurturing Care* e a atenção aos ODS, tendo em vista a escassez de dados relacionados aos problemas de desenvolvimento na infância. Na vigilância do desenvolvimento infantil, os cuidados de saúde da criança, as práticas parentais, a educação infantil e a promoção da saúde na atenção aos bebês e crianças pequenas são de grande relevância para alcançar o desenvolvimento adequado na primeira infância, requerendo investimentos e recursos^(1)^.

A enfermagem tem corresponsabilidade para fazer do contato com a criança e sua família uma janela de oportunidades, com intervenções à luz dos ODS^(7)^, diante da contagem regressiva para 2030, para contribuir com um ambiente facilitador, com proteção e segurança, e garantia de leis e políticas públicas eficazes^(1,3)^. A vigilância do desenvolvimento infantil representa, portanto, ação promissora para detectar problemas, compreender dificuldades e buscar atender às necessidades de saúde para a promoção e proteção na primeira infância.

Os cuidadores parentais têm muitas tarefas para exercer o cuidado e compreender informações, que podem ser complexas, novas, específicas e/ou incertas, sendo essencial para a infância os conhecimentos parentais sobre o desenvolvimento infantil e seus benefícios para o cuidado integral da criança^(9)^. O diálogo com enfermeiras(os), a exemplo do cuidado de enfermagem em VD, é considerado por cuidadores parentais favorecedor de abertura e sensibilidade, identificador de necessidades e esclarecedor de dúvidas, em uma perspectiva de envolvimento e participação^(4)^. Estudo aponta que, por meio de uma prática contextualmente relevante, equitativa e acessível de atenção às necessidades de crianças e adolescentes, as(os) enfermeiras(os) contribuem, sobremaneira, para o progresso do alcance dos ODS, mesmo diante de muitos desafios^(7)^.

Em termos de formação em enfermagem, as oportunidades durante o curso de graduação são imprescindíveis para constituir bases fortes e potencialidades para a oferta de práticas qualificadas. As experiências de aprendizagem materno-infantil no campo da APS são importantes para estudantes de graduação, particularmente em ambientes simulados e clínicos. A simulação tem peculiaridades para complementar a aprendizagem, sendo apontada como preparação que aumenta a confiança na prática, e as experiências de estudantes de enfermagem em simulação de atenção à saúde materno-infantil com *debriefing* estruturado se mostraram eficazes no aprendizado^(6)^. Os ambientes clínicos de APS são considerados oportunidades valiosas de aprendizagem para estudantes de enfermagem, contribuindo para obtenção de competências e desenvolvimento de resiliência, sendo importante garantir a preparação do campo no qual a(o) estudante será inserida(o), o acolhimento da equipe e a supervisão próxima^(10)^.

Por outro lado, há que ressaltar as dificuldades para o acesso universal aos serviços de saúde diante das dificuldades na prestação de cuidados de saúde infantil avançado relacionadas à escassez de profissionais de saúde qualificados^(7)^. Assim, cabe maior integração das metas dos ODS tanto na formação quanto na educação permanente de enfermeiras(os), buscando viabilizar uma prática clínica qualificada em vários contextos e ambientes, com maior atenção à justiça social, saúde ambiental e construção de parcerias^(5)^. Desse modo, abrem-se possibilidades para, ao mesmo tempo, contribuir com a Agenda 2030 e ampliar a qualificação do perfil profissional da(o) enfermeira(o).

### Limitações do estudo

Na explanação da presente perspectiva educacional, não foram pormenorizadas todas as dimensões teórico-práticas que as(os) estudantes vivenciam durante a disciplina de graduação ora apresentada. Também não foi sistematizada uma avaliação dos estudantes com relação **à** proposta educacional, sendo importante o *feedback* de todos os envolvidos para retroalimentar os caminhos pedagógicos, cabendo avançar e incrementar estudos sobre as competências e processos de ensino-aprendizado.

### Contribuições para as áreas da enfermagem e saúde

A perspectiva educacional apresentada aponta a relevância da primeira infância como fase essencial para o cuidado em saúde da criança, quando é mais eficaz agir e menos dispendioso do que buscar posteriormente reverter ou mitigar os efeitos das adversidades.

No presente relato, o incremento de estratégias educacionais e o aprimoramento dos conhecimentos foram importantes para trazer elementos qualitativos a favor do aprendizado e discussão sobre a primeira infância e a vigilância do desenvolvimento infantil. Esse movimento contribui para o cuidado de enfermagem, particularmente em ambientes de cuidados primários de saúde da criança, consoante aos preceitos dos ODS, com destaque aos objetivos 1, 3, 4 e 10.

## CONSIDERAÇÕES FINAIS

A presente perspectiva educacional destacou a vigilância do desenvolvimento infantil na formação de enfermeiras(os) como uma triangulação entre saberes teóricos, simulação e ambiente clínico.

No caminho apresentado, a ênfase é proporcionar vivências nas situações simuladas e práticas junto às famílias com crianças em VD, detalhando experiências durante a formação em enfermagem entre pares e aprendendo a reconhecer fortalezas, fragilidades e potencialidades das práticas existentes e as barreiras a serem rompidas. Tais aspectos são importantes para a visão de diferentes contextos e circunstâncias diante dos desafios contemporâneos para alcance dos ODS.
